# Viewpoint on the review by Savioli and colleagues on the 2017 WHO guideline on soil-transmitted helminth infections in at-risk population groups

**DOI:** 10.1371/journal.pntd.0006383

**Published:** 2018-04-26

**Authors:** Antonio Montresor, Juan Pablo Peña-Rosas, Pura Rayco-Solon, Francesco Branca, Susan L. Norris, Gautam Biswas

**Affiliations:** 1 Control of Neglected Tropical Diseases, World Health Organization, Geneva, Switzerland; 2 Nutrition for Health and Development, World Health Organization, Geneva, Switzerland; 3 Information, Evidence and Research, World Health Organization, Geneva, Switzerland; University of Kelaniya, SRI LANKA

## Dear PLOS editor

Thank you for sharing the review by Savioli and colleagues [1] on *Preventive chemotherapy to control soil-transmitted helminth infections in at-risk population groups* [2] and for giving us the opportunity to clarify several important points about the preparation and the aim of this World Health Organization (WHO) guideline.

Several Member States, partners, and other units in WHO requested that the Departments of Control of Neglected Tropical Diseases and Nutrition for Health and Development assess all the evidence supporting the recommendations issued by the Organization on deworming.

A guideline review [3]—the formal WHO process to assess the existing evidence—was applied on deworming: Guidelines published by WHO consist of a comprehensive and objective review of the evidence relevant to key areas of uncertainty in a given field and of an explicit and transparent process for formulating recommendations.

A Guideline Development Group (GDG) on soil-transmitted helminth infections (STHs) was established and included 16 experts, external to WHO, with different areas of expertise, perspectives, and geographical origin [2].

The GDG reviewed all available evidence, including the recent Cochrane and Campbell reviews [4–6] and other systematic reviews on STH morbidity, drug efficacy, drug safety, and the cost of different logistic approaches for drug administration [7–11]. The GDG concluded that the existing WHO recommendations for preventive chemotherapy in the three groups at risk are still valid. Quality of evidence was assessed with the Grading of Recommendations Assessment, Development, and Evaluation (GRADE) approach [12].

Several of the five critical areas in which this WHO guideline ‘does not provide the necessary leadership’, according to Savioli and colleagues [1], include practical aspects of programme implementation that are addressed in separate WHO manuals focused on implementation, monitoring, and evaluation (e.g., monitoring for drug coverage and resistance).

We agree that programme managers need to continue to use existing practical manuals since WHO recommendations on deworming remain unchanged. These manuals include those on controlling STHs, achieving universal access to safe water and adequate sanitation and hygiene interventions, and addressing all forms of malnutrition.

The ‘critical contemporary topics’ mentioned by Savioli and colleagues [1] are in part outside the scope of these guidelines (e.g., defining endemicity thresholds and groups at risk for preventive chemotherapy for other NTDs) and in part articulated in the ‘research gaps’ chapter of the guidelines (e.g., alternative anthelminthic medicines or combinations, innovative distribution system to reach adolescent girls). The GDG considered them important areas in which additional evidence should be collected before WHO can issue recommendations. WHO technical units and our collaborating centres are actively working on these topics.

We thank the authors of the review for their attention to the work of WHO and for their broad agreement with the conclusions of this guideline. While we recognise that they have concerns with the widely accepted methods used by WHO to develop evidence-informed guidelines, we nonetheless look forward to working with all of our partners to decrease the global burden of STHs and other neglected tropical diseases.

## Notice

In any use of this article, there should be no suggestion that WHO endorses any specific organisation, products, or services. The use of the WHO logo is not permitted. This notice should be preserved along with the article’s original URL.
